# Directly Electrospun Carbon Nanofibers Incorporated with Mn_3_O_4_ Nanoparticles as Bending-Resistant Cathode for Flexible Al-Air Batteries

**DOI:** 10.3390/nano10020216

**Published:** 2020-01-27

**Authors:** Ying Yu, Yuxin Zuo, Ying Liu, Youjun Wu, Zhonghao Zhang, Qianqian Cao, Chuncheng Zuo

**Affiliations:** 1 College of Mechanical and Electrical Engineering, Jiaxing University, Jiaxing 314000, China; yingyu@mail.zjxu.edu.cn (Y.Y.); wuyoujun@outlook.com (Y.W.); zhonghaozhang2019@hotmail.com (Z.Z.); qqcao@mail.zjxu.edu.cn (Q.C.); 2 College of Design, Jiaxing University, Jiaxing 314000, China; liuying@mail.zjxu.edu.cn; 3 School of Mechanical and Aerospace Engineering, Jilin University, Changchun 130025, China

**Keywords:** bending-resistant cathode, electrospinning, flexible Al-air batteries

## Abstract

Al-air batteries are regarded as potential power source for flexible and wearable devices. However, the traditional cathodes of Al-air batteries are easy to be broken after continuous bending. This is why few Al-air batteries have been tested under the state of dynamic bending so far. Herein, carbon nanofibers incorporated with Mn_3_O_4_ catalyst have been prepared as bending-resistant cathodes through direct electrospinning. The cathode assembled in Al-air battery showed excellent electrochemical and mechanical stability. A high specific capacity of 1021 mAh/cm^2^ was achieved after bending 1000 times, which is 81.7% of that in platform state. This work will facilitate the progress of using Al-air battery in flexible electronics.

## 1. Introduction

Flexible and wearable electronic devices have enjoyed rapid development in recent years [1,2,3]. The wearable devices may be placed on wrists as pulse sensors for health monitoring [4], or on legs as muscle sensors [5] for rehabilitation exercises. The supplied power sources should remain stable during the dynamic bending caused by muscle contractions. As the next-generation energy storage devices, Al-air batteries are attracting great interest owing to their high theoretical electrochemical equivalent, environmental friendliness and good safety [6,7,8,9,10] Air cathode is an essential and vulnerable part of the Al-air battery. The traditional preparation of cathodes requires physical deposition of all ingredients onto current collectors by drop-casting or spraying-coating [11,12,13]. However, this kind of cathodes are easy to damage during the process of repeated bending, which would make them impractical for flexible electronic devices.

To address this problem, electrospinning has been explored as a novel method to prepare flexible cathodes [14,15,16,17,18]. Carbon nanofibers (CNFs) could be prepared through electrospinning followed by heat treatment. Catalysts are added on the surface of CNFs by physical adsorption or chemical growth [13,14,18,19,20]. Zhang et al. [6] presented a brief introduction to the recent development of flexible air cathodes for metal-air batteries. Wang et al. [16]. fabricated cathodes by electrospinning CNFs and employed iron carbide as catalyst for flexible Al−air batteries. Cao et al. [21] reported the preparation of CNFs covered with Co_9_S_8_ nanoparticles as cathodes for Al-air batteries. However, most studies mainly focused on the electrochemical performance of Al-air batteries under static state. Zhong et al. [22] presented a flexible Zn-air battery and tested the electrochemical performance under dynamic stretching conditions. Han et al. [17] studied the discharge characteristics of Zn-air batteries under static bending state with different bending angles. To the best of our knowledge, few studies have focused on the electrochemical performance of Al-air batteries under dynamic bending state, which is really important to their application in flexible and wearable devices.

In this work, we propose an innovate bending-resistant cathode for Al-air battery prepared by direct electrospinning. Manganese materials are served as catalyst. Instead of the conventional chemical post-treatment, wet-milling is introduced to solve the problem of insoluble manganese dioxide particles. All cathode materials are mixed uniformly and electrospun all-in-one step. Flexible Al-air batteries are assembled with the as-fabricated cathode. The as-fabricated cathodes possess the advantages of high porosity and large specific surface area, which would contribute the oxygen diffusion from outside and benefit the performance of batteries [15,23,24]. The electrochemical performance of the Al-air battery has been studied under the static and dynamic bending states of platform. The results showed that the Al-air batteries have perfect electrochemical performance and excellent mechanical flexibility. The cathodes have good resistance to continuous bending. The current study is of great significance for the applications of Al-air batteries in flexible and wearable electronic devices.

## 2. Materials and Methods

### 2.1. Materials

All materials in this study were of analytical grade and used as received without further treatment. Polyacrylonitrile (PAN, Mw = 150000 g/mol) and N,N-dimethylformamide solution (DMF, AR, 99.5%) were obtained from Macklin Biochemical Co., Ltd. (Shanghai, China). MnO_2_ particles were purchased from Kejing Co., Ltd. (Shenyang, China). Zirconia balls were obtained from Nikkato Co., Ltd. (Osaka, Japan).

### 2.2. Preparation of Air Cathmodes

**Figure 1:**
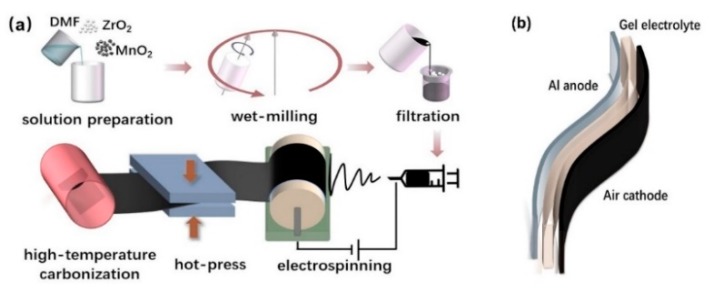
(**a**) Schematic illustration of the fabrication of air cathodes by directly electrospinning. (**b**) Sandwich structure of the Al-air battery.

MnO_2_ nanoparticles were prepared using a rotation/revolution mixer (NP-100, Thinky Co., Ltd., Tokyo, Japan) equipped with cooling unit. 7 mL DMF, 4 g MnO_2_ and 10 g zirconia balls with a diameter of 0.1 mm were put into the vessel of mixer. MnO_2_ suspension was nanosized by 3-step wet-milling with pulverizing conditions as illustrated in Figure 1a: the first step (pulverization process), 2000 rpm for 2 min and chill for 5 min. This process was repeated for 5 times. The second step (dispersion process), 500 rpm for 2 min after addition of 3 mL DMF. The third step (separation process), remove the 0.1 mm-diameter zirconia balls from the suspension. The precursor solution for electrospinning was prepared by dissolving 1 g PAN in the above suspension. To obtain a homogeneously mixed solution for electrospinning technique, magnetic stirring was applied for 6 h at 60 °C.

Then, the precursor solution was electrospun via a single-capillary electrospinning apparatus. The tip-collector distance, applied voltage and flow rate were fixed at 15 cm, 17 kV and 1.2 mL/h, respectively. The electrospun fibers were collected on a rotating drum covered with nickel foam. The obtained fibers were dried in ambient air for 24 h and then hot-pressed with 200 Mpa at 230 °C for 30 min. The films were carbonized at 900 °C for 1 h in argon atmosphere with a heating rate of 5 °C/min. The fabrication procedure of the cathodes is shown in Figure 1a.

### 2.3. Characterization

The morphological structure of the electrospun films was investigated through SEM (Su-8010, Hitachi Co., Ltd., Tokyo, Japan). The crystalline structures of the samples were characterized using an X-ray diffraction (XRD) instrument (Bruker D8 Advance, Bruker Corp., Billerica, MA, USA) with a Cu kα radiation of 0.1541 nm as an X-ray source. Raman spectra were measured using an Invia Raman Microscope (Invia Microscope Co., Ltd., Renishaw, U.K.). X-ray photoelectron spectra (XPS) of the samples were conducted using ESCALAB 250Xi (Thermo Fisher Scientific, CA, USA) instrument. Nitrogen adsorption–desorption analysis was carried out by using Smart Sorb 92/93 surface area analyzer.

Solid Al-air batteries were assembled with the sandwich type as schematically illustrated in Figure 1b. The solid-state polymer alkaline gel electrolyte was prepared according to a previous report [25]. A 4 × 3 cm aluminum foil was served as the metal anode. The constant current discharges were carried out using a battery testing system (CT2001A, LAND Electronics Co., Ltd., Wuhan, China). Electrochemical impedance spectroscopy (EIS) measurements were tested by an electrochemical workstation (RST5000, Shiruisi Co., Ltd., Zhengzhou, China) with an AC amplitude of 10 mV and frequency from 0.01 Hz to 100 kHz. The activity of the catalysts towards the oxygen reduction (ORR) was evaluated by linear sweep voltammetry (LSV) from 1.0 to 0.2 V vs. RHE at a scan rate of 5 mV/s and 1600 rpm in a O_2_-saturated 0.1 M KOH solution. The reference electrode and counter electrode were Hg/HgO and platinum wire.

## 3. Results

### 3.1. Characterization of the Cathodes

**Figure 2:**
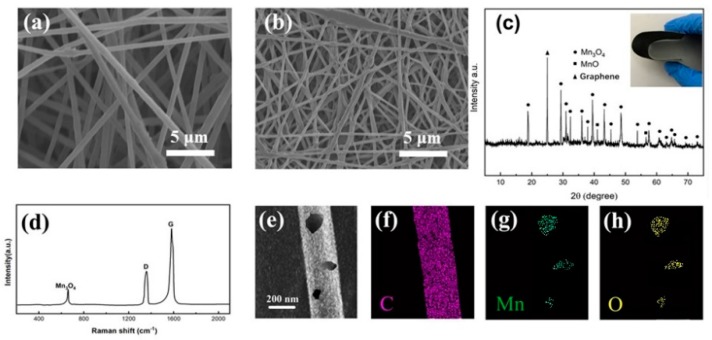
SEM images of the electrospun fibers before (**a**) and after (**b**) heat treatment. (**c**) XRD patterns of the nanofibers on cathodes. (**d**) Raman spectra of Mn_3_O_4_/CNFs. (**e**–**h**) Element mappings of Mn_3_O_4_/CNFs.

PAN and MnO_2_ were co-electrospun to form nanofibers. After heat treatment, Mn_3_O_4_ were obtained and encapsulated in CNFs. The direct contact between the active material and the current collector was favorable for electron transport and the kinetics were improved [6]. The electrospun films before heat treatment exhibited a smooth surface and nearly straight fiber morphology with uniform diameters of ~400 nm as shown in Figure 2a. After heat treated (hot-pressed and carbonization), the surface of the sample became a little rougher due to the decomposition of organic components of PAN [26], but it retained well-defined fibrous morphology with a diameter of ~250 nm as shown in Figure 2b.

The crystal structure of Mn_3_O_4_ was confirmed by XRD patterns in Figure 2c. Peaks at 2θ = 41° and 2θ = 56° were ascribed to the residue presence of manganese oxide coming from the hot-press process. A sharp peak at 2θ = 26.4 indicated a graphitic structure of CNF within the samples [27]. The other characteristic diffraction peaks observed in the pattern of the nanofibers represent the reflections of Mn_3_O_4_. Raman spectrum was recorded, and the corresponding result is shown in Figure 2d. The Raman peaks centering around 1365 and 1584 cm^−1^ can be ascribed to the D and G bands of carbon, respectively. The D band corresponds to the defects or disorder of carbon, while the G band is indicative of ordered carbon [28,29]. The ratio of I_D_/I_G_ is 0.85, indicating that there are few defects in the crystalline structure [30]. The peak at 651 cm^−1^ is characteristic of the Mn-O vibrational mode, proving that the Mn_3_O_4_ nanoparticles were successfully doped in the CNFs [31]. Element mappings (Figure 2e–h) in a single nanofiber further show that most Mn and O are uniformly distributed in the nanofibers.

**Figure 3:**
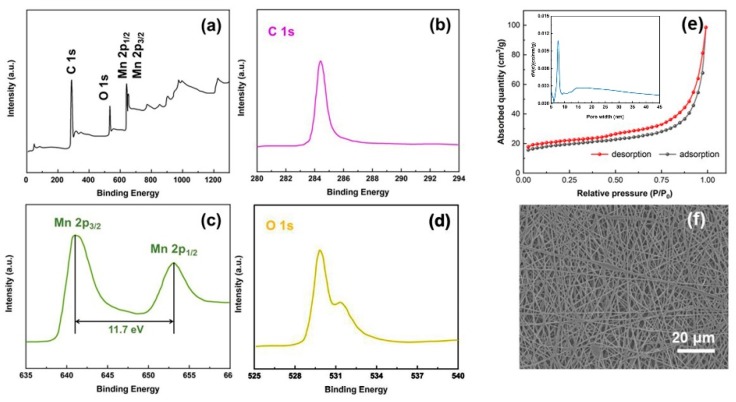
XPS spectra: (**a**) wide-scan and narrow-scan at (**b**) C 1s region, (**c**) Mn 2p region, and (**d**) O 1s region for the as-prepared Mn3O4/CNFs. (**e**) N_2_ adsorption/desorption isotherm of the Mn_3_O_4_/CNFs sample and their pore size distribution curve (inserted). (**f**) SEM photographs of the Mn_3_O_4_/CNFs.

XPS measurements were conducted to investigate the chemical composition as well as the bonding state of the Mn_3_O_4_/CNFs. The overall XPS spectrum of the Mn_3_O_4_/CNFs composite reveals the coexistence of the elements Mn, O and C clearly in Figure 3a. The narrow-scan XPS spectra of Mn 2p, O 1s and C 1s after Gaussian fitting are shown in Figure 3b–d, respectively. Two obvious peaks in Mn 2p spectra are located at 641.2 and 652 eV, which are attributed to the characteristic Mn 2p_3/2_ and Mn 2p_1/2_ spin-orbit states of Mn_3_O_4_. The splitting width of 11.7 eV is in good agreement with those reported for Mn_3_O_4_ [32]. The O 1s XPS spectra exhibit two peaks at 529.8 and 531.5 eV, corresponding to the Mn-O-Mn bond in the oxide and Mn-O-H hydroxide [33]. The C 1s has only one intensive peak at 284.5 eV due to C-C sp^2^ bonding for all the materials [34]. The isotherms of N_2_ adsorption-desorption for the Mn_3_O_4_/CNFs are shown in Figure 3e. The adsorption-desorption isotherm curve of the composites can be classified as a type-IV curve with hysteresis loop of 0.3–1.0 p/p_0_, suggesting the porous structure of the as-fabricated Mn_3_O_4_/CNFs. The Brunauer–Emmett-Teller (BET) surface area is measured to be about 42.5 m^2^/g. The pore-size distribution (the inset of Figure 3e) of the Mn_3_O_4_/CNFs centers at ~7.9 nm. The three-dimensional network structure of the as-fabricated air cathode is shown in Figure 3f from a mesoscopic view.

### 3.2. Electrochemical characterization of Al-Air batteries

**Figure 4:**
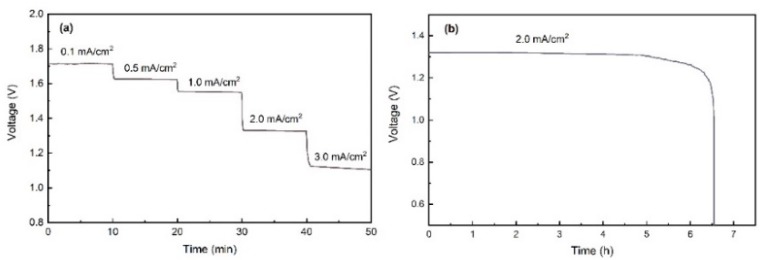
(**a**) Rate discharge curves of Al-air battery at flat state. (**b**) Galvanostatic discharge curve of Al-air battery at 2 mA/cm^2^ with flat state.

Figure 4 shows the rate discharge performance of Al-air batteries at various current densities (from 0.1 to 3.0 mA/cm^2^). The voltage plateaus are stable at 1.72 to 1.08 V with increasing current densities, suggesting that the Al-air batteries exhibit a good stability over a wide range of discharge currents. Discharge curves of Al-air battery at 2.0 mA/cm^2^ are given in Figure 4b. The specific capacity is calculated as 1273 mAh/cm^2^, which is closed to the value (1287.3 mAh/cm^2^) reported in the previous work of flexible Al-air battery [16].

**Figure 5:**
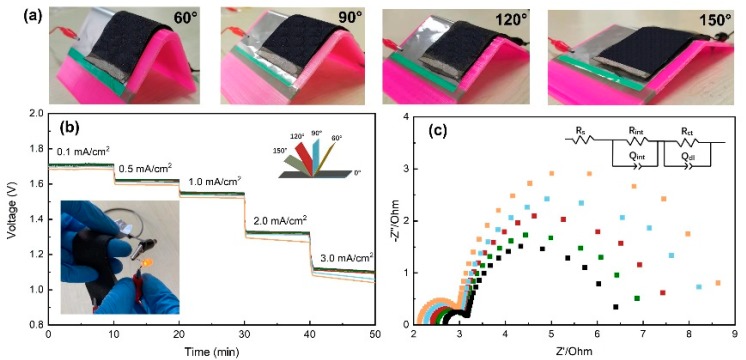
(**a**) Al-air batteries at different bending angles. (**b**) Rate discharge profiles and (**c**) EIS plots for Al-air batteries at different bending angles.

To further investigate the mechanical flexibility, the Al-air batteries are intentionally bended with different angles as shown in Figure 5a. Figure 5b shows the rate discharge performance of the batteries at different bending angles (platform, 60, 90, 120 and 150°). The discharge voltage remains almost unchanged at bending angles of 120 and 150°. While the discharge voltage plateaus decrease obviously at high current densities when decreasing the bending angle to 90 and 60°, which is similar to the study reviewed by Liu et al. [6]. For instance, the discharge voltage is 1.01 V at the state of 60° bending at a current density of 3.0 mA/cm^2^. There is about 12% more decrease than that in platform state (1.15V). In order to study the effects of bending angles on the discharge property, EIS measurements were performed at different conditions. The Nyquist plots as shown in Figure 5c consist of two semicircles in the high and low frequency regions, which can be fitted to the given equivalent circuit model. The circuit consists of five elements: solution resistance (R_s_), resistance at the electrode/electrolyte interface (R_int_), resistance of the charge transfers during the electrochemical process (R_ct_) and constant phase elements associated with the capacitances arising at the electrode/electrolyte interface (Q_int_ and Q_dl_). The resistance components are summarized in Table 1. Remarkably lower resistance values are seen for batteries at flat state compared with that of the batteries at different bending state. The low value R_int_ for the flat state is associated with the fully contact between the electrolyte and electrode. The intimate contact support providing a facile electron pathway, and leading to a faster reaction. In contrast, the bending area of the battery in a bending state will bear a certain bending tension, which will cause slight displacement at the electrode/electrolyte interface in other areas and increase the contact resistance. Reducing the bending angle is equivalent to reducing the bending radius, which would increase the bending tension and cause more displacement [35]. This explains why the contact resistance increases as the bending angle decreases. As shown in Figure 5c, Al-air battery exhibits the smallest resistance R_int_ and R_ct_ at platform state, suggesting that the bending state relatively decreased the conductivity of the batteries. A similar phenomenon was also found in the study of Peng [17] for flexible Zn-air battery.

**Figure 6:**
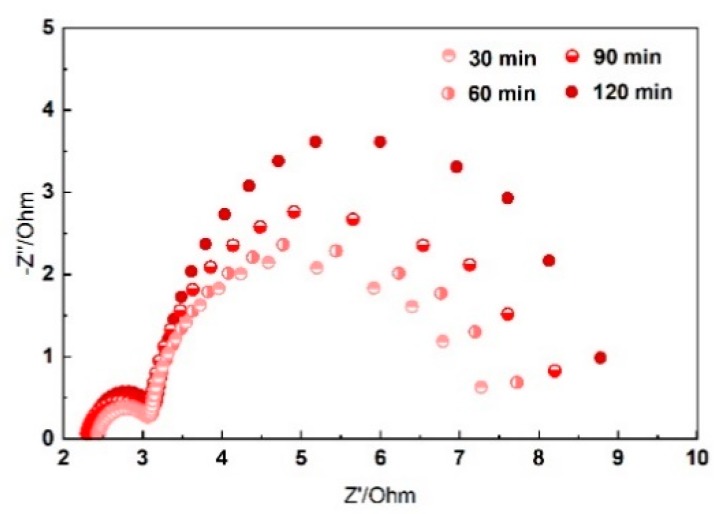
Impedance spectra presented in Nyquist plots of Al-air batteries as a function of time.

The impedance spectra of Al-air batteries as a function of time are plotted in Figure 6 at static bending state. Take 120° bending as an example; the EIS behavior of the system can be characterized by a high frequencies capacitive loop related to the charge transfer process owing to the dissolution of Al anode and a second capacitive loop at low frequencies due to the growth of side reaction products [36]. The diameter of the capacitance loops is increased as a function of time. Al(OH)_3_ is the main side reaction product during the electrochemical reaction. Since Al^3+^ ions are thermodynamically unstable, Al(OH)_3_ are formed when Al comes into contact with alkaline electrolyte. The generated Al(OH)_3_ are difficult to dissolve in the solid electrolyte and will accumulate at the interface between the electrolyte and anode. For this reason, the charge transfers and inter resistance increase as a function of time. The electrochemical performance of the solid Al-air batteries decreased as the reaction proceeds. Effective control of Al corrosion in alkaline electrolyte is very important for flexible Al-air batteries.

**Figure 7:**
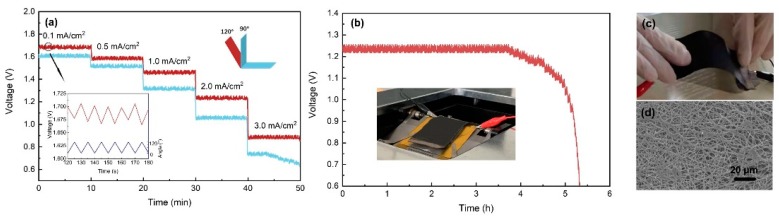
(**a**) Rate discharge profiles at dynamic bending state. (**b**) Galvanostatic discharge curve at 2 mA/cm^2^ under 120° dynamic bending, and the inset is the device for dynamic bending test. Photograph (**c**) and SEM (**d**) of the main bending area of the Al-air battery after bending for 1000 times.

Figure 7a shows the discharge curves of the Al-air batteries under continuously dynamic bending (90 and 120°) state. The batteries complete a bending in the span of 10 s. The voltage plateaus decreased obviously at 90° bending, and this might be attributed to the loose contact between the electrodes and electrolyte. As can be seen from the inset in Figure 7a, the variation of the voltage during the bending is closely related to the bending frequency. The peak and valley values of the voltage appeared when the batteries were at the state of platform and 120° bending; we thought that there may be a slight separation between the electrodes and electrolyte. Figure 7b demonstrates the continuous discharge of the Al-air battery at 2 mA/cm^2^ under 120° dynamic bending state. The discharging time is 5.29 h, and the corresponding specific capacities is 1021 mAh/cm^2^, which is 81.7% of that under platform state. After continuous bending for 1000 times, the air cathode is not broken in a macroscopic view as shown in Figure 7c. We observed the bending area of the air cathode with SEM in Figure 7d, and no cracks are detected in the three-dimensional network structure. It proves that the cathode is resistant to the repeated bending.

**Figure 8:**
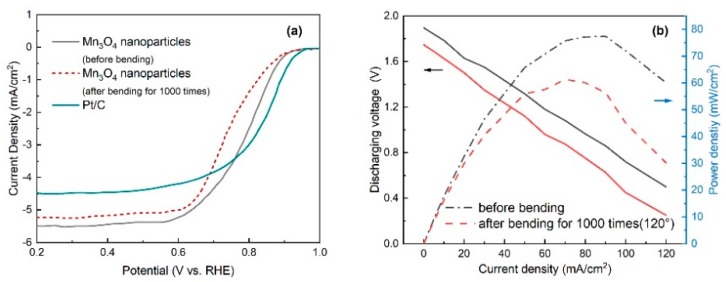
(**a**) ORR polarization curves of the air cathodes and (**b**) discharging voltage/power density profiles before and after bending for 1000 times.

In order to evaluate the behavior of the catalysts in ORR before and after bending for 1000 times, the electrochemical performance of the air cathodes towards the ORR are investigated by half-cell testing at a rotation rate of 1600 rpm. As can be seen from Figure 8a and Table 2, the electrode before bending exhibits high electrocatalytic activity toward ORR with a comparable onset potential of 0.92 V to that of commercial 20% Pt/C (0.95 V), along with a higher limiting current density of 5.52 mA/cm^2^ than that of Pt/C (4.49 mA/cm^2^), indicating the promising ORR properties. By contrast, the sample after bending for 1000 times exhibits the lower activity with the ORR onset potential of 0.83 V, while the low activity of the cathode could be attributed to the hydroxide precipitate. The hydroxide precipitate is formed during the electrochemical reaction and is difficult to dissolve in the solid electrolyte. The hydroxide precipitate decreases the specific surface areas and reduces the active sites’ exposure [37]. The galvanodynamic discharge curves and the corresponding power densities of the Al-air battery are further studied after 120° bending for 1000 times as shown in Figure 8b. The maximum power density of the battery is 56.5 mW/cm^2^ after bending for 1000 times, slightly lower than that without bending test (75.5 mW/cm^2^). The excellent performance could be attributed to the interconnected open-pore microstructure of cathode and the encapsulated catalyst; the free space between constituent fibers can attenuate the bending stress effectively without damaging the fibers [38].

## 4. Conclusions

In summary, bending-resistant cathodes have been successfully developed by direct electrospinning for highly flexible Al-air batteries. The results showed that the electrochemical performance is slightly affected by the bending state, and this could be attributed to the higher internal resistance due to the separation between the electrodes and electrolyte during the bending. The battery can be discharged over 1.2 V under 2 mA/cm^2^ at dynamic bending state, and the specific capacity could reach up to 1021 mAh/cm^2^. The assembled Al-air batteries with the bending-resistant cathodes exhibit excellent electrochemical and mechanical performance. This study has great potential for the application of Al-air batteries in wearable energy-storage devices.

## Figures and Tables

**Table 1:** The resistance values of the equivalent circuit elements based on EIS measurements of Al-air batteries.

Element	Flat	150°	120°	90°	60°
R_int_ (Ω)	0.56	0.62	0.75	0.87	0.98
R_ct_ (Ω)	2.64	2.95	3.12	4.63	5.21

**Table 2:** ORR activities and kinetics for different catalysts.

Catalyst	Onset Potential (V vs. RHE)	Limiting Current Density @1600 rpm (mA/cm^2^)	Electron Transfer Number (n)
Mn_3_O_4_ nanoparticles (before bending)	0.92	5.52	4.1
Mn_3_O_4_ nanoparticles (after bending for 1000 times)	0.83	5.26	3.6
Pt/C	0.95	4.49	4.0
